# Simple steps to equity in child survival

**DOI:** 10.1186/1741-7015-11-261

**Published:** 2013-12-18

**Authors:** Stuart Gilmour, Kenji Shibuya

**Affiliations:** 1 Department of Global Health Policy, Graduate School of Medicine, University of Tokyo, Hongo 7-3-1, Bunkyo-ku, Tokyo 113-0033, Japan

**Keywords:** Breastfeeding, Child mortality, Health inequity, Interventions, Millennium development goals

## Abstract

Although the number of child deaths has declined globally over the past 20 years, many countries still lag behind their millennium development goal targets, and inequity in child health remains a pernicious problem both between and within countries. Breastfeeding is a key intervention to reduce child mortality, and in an article published in *BMC Medicine,* Roberts and colleagues have shown that breastfeeding interventions can have a significant role in reducing inequity in child health. With the proper attention paid to overcoming the barriers to scaling up breastfeeding interventions, deployment of effective interventions in health facilities and the community, and improvements in support for breastfeeding interventions across society, many countries that are struggling to meet their millennium development goals could make significant gains in child survival and inequity.

Please see related research: http://www.biomedcentral.com/1741-7015/11/254/abstract.

## Background

Significant progress has been made towards achieving Millennium Development Goal 4 (MDG 4), which pertains to reducing child mortality, but much more is still to be done [1]. Since the turn of the millennium the number of child deaths has declined significantly, from an estimated 11.6 million in 2000 to 7.2 million in 2010 [2]. However, greater efforts are required if the world is to meet the millennium development goals [2], and despite recognition of the problem, inequity in child health remains a persistent and galling issue holding back progress [3]. Although the importance of within-country inequalities has been recognized, they are difficult to eliminate. Interventions to improve accessibility and coverage of health services are often taken advantage of first and most successfully by the wealthiest segments of society [4]. Further, interventions such as cash transfers that target welfare and poverty directly may not have observable health benefits [5].

It is in this context that Roberts *et al*. show the potential for breastfeeding interventions to reduce inequity in child mortality [6]. Roberts and colleagues use available data to estimate the changes in prevalence of exclusive and partial breastfeeding in 137 developing countries, separately by wealth quintile, and show that gains in breastfeeding coverage are equal across wealth quintiles. Breastfeeding does not rely on health infrastructure, is not taken up preferentially by the wealthy, and helps to prevent diseases such as pneumonia that have higher prevalence in poorer communities [7]. Therefore, breastfeeding interventions have greater potential than others to reverse major inequalities in child mortality [8]. Breastfeeding also plays an important role in addressing both the short-term and long-term effects of malnutrition, and can have greater benefits in the poorest communities [9].

For the benefits of breastfeeding to be realized, however, rates of breastfeeding need to be high in all countries, and Roberts *et al*. present a mixed picture of success in this regard. Some countries have made remarkable progress in scaling up breastfeeding as a child health intervention since 1990: in Malawi, for example, rates of exclusive breastfeeding in the first 5 months of life have increased from 5.0% in 1990 to 49.7% in 2010, and over this time period the rates of predominant breastfeeding in some countries have doubled from a low base. However, many countries, often those with the highest rates of child mortality, have regressed during this time. In some countries, concerns about mother-to-child transmission of HIV are likely to contribute to low rates of breastfeeding [10], despite strong World Health Organization (WHO) guidelines on HIV and infant feeding practices [11]. In these countries, better understanding of the competing risks of suboptimal breastfeeding and HIV/AIDS, better adherence to WHO guidelines, and education of mothers and health providers, are essential to improve breastfeeding rates in future. However, some countries with low HIV prevalence such as Afghanistan, Guyana and Indonesia have shown a reduction in exclusive breastfeeding rates over the duration of the study [6]. Understanding the barriers to breastfeeding in these countries, and identifying interventions that will work, is crucial to reducing child mortality and health inequity.

What is to be done to encourage breastfeeding in these countries? What interventions are complementary to and support breastfeeding, and what intersectoral gains need to be made to support breastfeeding interventions in those countries that are furthest from achieving MDG 4?

### Interventions to support breastfeeding

The efficacy of basic interventions to promote breastfeeding is well established [12], and the benefits of an ambitious scale up of breastfeeding in developing nations potentially substantial [13]. Breastfeeding interventions can also be implemented without substantial investment in facilities and medical technology or other medical infrastructure, and offer the potential for a cheap, high coverage mechanism to elevate the health of all children, without leaving the poorest behind. However, effective interventions often involve peer education, individual counseling and prenatal and postnatal support. These are interventions that depend on the availability of a workforce that is often lacking in countries most in need of interventions to scale up breastfeeding. Furthermore, these interventions can lose their efficacy as they are scaled up and incorporated into standard health system structures [14]. As is the case with most effective community health interventions, counseling interventions administered by community health workers require sufficient remuneration, training and workplace support [15]. Such conditions do not necessarily exist in low-income countries where prenatal counseling and support is most needed, and will need to be established early in the process of expanding both breastfeeding-specific and broader maternal and child health (MCH) interventions. Without careful attention to the levels of payment, training and professional support that community health workers receive, it will be difficult to build a sustainable intervention capable of making the large-scale, prolonged and broad-based changes to breastfeeding practice necessary to achieve MDG 4.

### Beyond the health sector

Breastfeeding is also a nutrition intervention with significant developmental and welfare benefits. The success of breastfeeding programs is also tied to the quality of maternal nutrition, and to the level of social support for breastfeeding. Roberts *et al*. rightly indicate the role of legislative changes and the media in encouraging and supporting breastfeeding, and this shows the important role of intersectoral collaboration in building an environment supportive of the full benefits of breastfeeding. Legislative support for public breastfeeding, family friendly workplace policies, strict standards on the content and advertising of baby foods for complementary feeding should become commonplace and acceptable community-level interventions in low-income nations.

Because breastfeeding does not rely on technology investment or extensive health infrastructure, it is also amenable to community based and grassroots initiatives to improve uptake [16], and these initiatives should be implemented and supported wherever possible. These complementary efforts have been shown to be effective at improving breastfeeding adherence in low-income nations [17], and are particularly important in settings where facility-based births are the minority [18]: typically, settings where exclusive breastfeeding is likely to have the largest effect on child mortality and inequality in infant health and development outcomes. In communities where most births occur in the home, it is not enough to have child friendly hospitals; instead, we need intersectoral development programs to build child friendly communities.

## Conclusions

Roberts *et al*. have identified the powerful equity benefits of scaling up breastfeeding, and quantified its significant contribution to preventing illness and mortality in low-income and middle-income countries. With this knowledge, we can better prioritize funding and system organization both for improving child health and for reducing inequity in illness and mortality in some of the poorest countries in the world. Nonetheless, challenges to scaling up breastfeeding remain. Only through careful attention to what is known to be effective and cost-effective, coupled with strategic use of multisectoral agents and cooperation across society, can we realize the large benefits of breastfeeding’s unique contribution to reducing the burden of disease and inequity in disease distribution in low-income and middle-income countries. With the deadline for the MDGs approaching and many countries still lagging on the key indicators of child health, now is the time to redouble efforts to scale up this cheap, reliable and equitable intervention, and to achieve the promise of better and more equitable health made in 2000.

## Competing interests

The authors declare that they have no competing interests.

## Authors’ contributions

Both authors contributed to conception of the article. SG drafted the article. Both authors were involved in editing and revision of the manuscript and both agreed to its publication.
